# Genetic and structural validation of Aspergillus fumigatus UDP-N-acetylglucosamine pyrophosphorylase as an antifungal target

**DOI:** 10.1111/mmi.12290

**Published:** 2013-07-05

**Authors:** Wenxia Fang, Ting Du, Olawale G Raimi, Ramon Hurtado-Guerrero, Michael D Urbaniak, Adel F M Ibrahim, Michael A J Ferguson, Cheng Jin, Daan M F Aalten

**Affiliations:** 1 Division of Molecular Microbiology, College of Life Sciences, University of DundeeDundee, DD1 5EH, Scotland, UK; 2 Division of Biological Chemistry and Drug Discovery, College of Life Sciences, University of DundeeDundee, DD1 5EH, Scotland, UK; 3 The College of Life Sciences Cloning Team, College of Life Sciences, University of DundeeDundee, DD1 5EH, Scotland, UK; 4 State Key Laboratory of Mycology, Institute of Microbiology, Chinese Academy of SciencesBeijing, 100101, China

## Abstract

The sugar nucleotide UDP-*N*-acetylglucosamine (UDP-GlcNAc) is an essential metabolite in both prokaryotes and eukaryotes. In fungi, it is the precursor for the synthesis of chitin, an essential component of the fungal cell wall. UDP-*N*-acetylglucosamine pyrophosphorylase (UAP) is the final enzyme in eukaryotic UDP-GlcNAc biosynthesis, converting UTP and *N*-acetylglucosamine-1-phosphate (GlcNAc-1P) to UDP-GlcNAc. As such, this enzyme may provide an attractive target against pathogenic fungi. Here, we demonstrate that the fungal pathogen *Aspergillus fumigatus* possesses an active UAP (*Af*UAP1) that shows selectivity for GlcNAc-1P as the phosphosugar substrate. A conditional mutant, constructed by replacing the native promoter of the *A. fumigatus* *uap1* gene with the *Aspergillus nidulans* *alcA* promoter, revealed that *uap1* is essential for cell survival and important for cell wall synthesis and morphogenesis. The crystal structure of *Af*UAP1 was determined and revealed exploitable differences in the active site compared with the human enzyme. Thus *Af*UAP1 could represent a novel antifungal target and this work will assist the future discovery of small molecule inhibitors against this enzyme.

## Introduction

*Aspergillus fumigatus* is one of the most important human opportunistic fungal pathogens that is largely responsible for the increased incidence of invasive aspergillosis (IA) in immunocompromised patients (Latgé, 2001, Latgé, 1999, Krappmann, 2006). Although three classes of antifungal drugs have been licensed for the treatment of IA (Walsh *et al*., 2008), its mortality rate remains extremely high (50%; Brown *et al*., 2012) partly due to limited antifungal treatment options, low efficiency and rising resistance against current antifungal drugs. Therefore, a next generation of antifungal drugs is urgently needed. Like in other pathogenic fungi, the cell wall of *A. fumigatus* is essential for fungal growth and as a barrier against chemical and environmental challenges. It is composed mainly of chitin, glucans and mannoproteins (Bernard and Latgé, 2001). Enzymes involved in cell wall biosynthetic pathways represent ideal drug targets as the fungal cell wall is a unique structure not found in mammalian cells. The newest echinocandin-class drugs (Douglas, 2001) are the first and only antifungal compounds that target the fungal cell wall by blocking β-1,3-d-glucan synthase (Douglas, 2001).

Chitin, accounting for 7–15% of the cell wall dry weight (Gastebois *et al*., 2009), is a β(1,4)-linked homopolymer of *N*-acetylglucosamine (GlcNAc) residues and is synthesized from UDP-GlcNAc. In eukaryotes, UDP-GlcNAc biosynthesis starts from the metabolite fructose-6-phosphate (Fru-6P) followed by amination to glucosamine 6-phosphate (GlcN-6P), acetylation to *N*-acetylglucosamine-6-phosphate (GlcNAc-6P), isomerization to GlcNAc-1P and uridylation to the sugar nucleotide UDP-GlcNAc (Milewski *et al*., 2006). The last step is catalysed by UDP-GlcNAc pyrophosphorylase (UAP) utilizing the high-energy pyrophosphate bonds from the substrate UTP to yield UDP-GlcNAc. UAP also exists in prokaryotes, usually referred to as GlmU, an enzyme with both acetylation and uridylation activity (Mengin-Lecreulx and van Heijenoort, 1993 19941993; 1994). In contrast to prokaryotes, the eukaryotic UAP enzymes are not capable of catalysing the acetylation reaction and are usually divided into mammalian and non-mammalian UAPs. The crystal structures of *Homo sapiens* UAPs (AGX1 and AGX2) have been determined (Peneff *et al*., 2001) and consist of a pyrophosphorylation domain and two further domains of unknown function. UAP from *Candia albicans* (*Ca*UAP1) (Maruyama *et al*., 2007) is the only non-mammalian UAP for which a crystal structure has been reported and is structurally similar to AGX1.

UAPs have been reported to be essential in many species (Araujo *et al*., 2005; Schimmelpfeng *et al*., 2006; Tonning *et al*., 2006; Zhang *et al*., 2008; Arakane *et al*., 2011). For example, a null mutation of the *Saccharomyces cerevisiae uap1* gene resulted in aberrant morphology and lethality (Mio *et al*., 1998). UAP is also essential for the bloodstream forms of *Trypanosoma brucei* (Stokes *et al*., 2008). No drug-like inhibitors have been described for this class of enzyme in eukaryotes. Only recently, a small-molecular compound from high-throughput screening exhibited inhibition of the uridyltransferase activity of GlmU in *Haemophilus influenzae* (Mochalkin *et al*., 2008).

Here we genetically and structurally validate UAP in *A. fumigatus* (*Af*UAP1) as a potential antifungal target using a conditional inactivation mutant of *uap1* and by determining the crystal structure of *Af*UAP1. Comparison with the human enzyme revealed exploitable differences in the active site providing a basis for the future design of small molecule inhibitors for this enzyme class.

## Results and discussion

### *A. fumigatus* possesses an active UAP1 enzyme selective for GlcNAc-1P and inhibited by UTP

A BLASTp search with the *H. sapiens* and *S. cerevisiae uap1* sequences (GenBank: BAA31202.1 and BAA31203.1) revealed a putative *uap1* gene in the *A. fumigatus* genome. This gene, including two introns, was amplified from genomic DNA and the introns were subsequently removed by site-directed mutagenesis. The ORF of the *uap1* gene was cloned into pGEX-6P-1 and overexpressed as a GST fusion protein in *Escherichia coli*. Purification using glutathione sepharose beads followed by GST cleavage and size exclusion chromatography yielded 5 mg of pure *Af*UAP1 protein per litre of bacterial culture.

**Figure 1:**
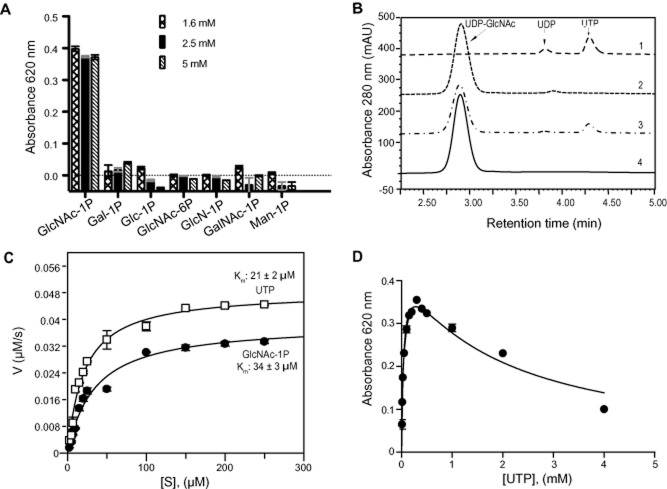
*Af*UAP1 enzyme activity assay.A. Substrate specificity against a range of phosphosugars. 100 nM of *Af*UAP1 was incubated with 2 mM UTP and 1.6 mM, 2.5 mM and 5 mM phosphosugars for 10 min and the products were analysed in a colorimetric Biomol green assay coupled with pyrophosphatase.B. HPAEC chromatogram for the *Af*UAP1 reverse reaction: 1, UTP standard; 2, UDP-GlcNAc standard; 3, 20 nM *Af*UAP1 in reaction; 4, no *Af*UAP1 in reaction.C. Representative determination of *K*_m_ for GlcNAc-1-P and UTP. The results are the mean ± SD for three determinations.D. Analysis of UTP substrate inhibition (*K*_i_ of 1.9 ± 0.2 mM) at concentrations >> *K*_m_. The results are the mean ± SD for three determinations.

The activity and substrate specificity of the *Af*UAP1 forward reaction were assessed by incubating the enzyme with UTP and various phosphosugars followed by analysing the products in a colorimetric assay coupled with pyrophosphatase. *Af*UAP1 has a preference for GlcNAc-1P as the phosphosugar substrate (Fig. 1A) as does *T. brucei* UAP1 (Stokes *et al*., 2008). In contrast, *Hs*UAPs (AGX1 and AGX2) recognize either GalNAc-1P or GlcNAc-1P to synthesize UDP-GalNAc or UDP-GlcNAc respectively (Wang-Gillam *et al*., 1998; Peneff *et al*., 2007).

*Af*UAP1 is also capable of catalysing the reverse reaction, conversion of UDP-GlcNAc and pyrophosphate (PPi) to UTP and GlcNAc-1P, as shown by the depletion of UDP-GlcNAc and the direct formation of UTP using HPAEC (Fig. 1B). Catalysis of this reaction has previously only been reported for *Hs*UAPs (Peneff *et al*., 2007).

The coupled colorimetric assay was used to measure the *K*_m_ values for GlcNAc-1P and UTP. *Af*UAP1 has a *K*_m_ of 34 ± 3 μM and 21 ± 2 μM for GlcNAc-1P and UTP, respectively (Fig. 1C, Table 1), comparable to *Hs*UAPs [AGX1, 5.3 and 53 μM and AGX2, 6.0 and 49 μM respectively (Peneff *et al*., 2007)]. However, the catalytic efficiency of *Af*UAP1 is much lower than *Hs*UAPs (Table 1). Interestingly, *Af*UAP1 activity is inhibited by UTP at a concentration above 1 mM (Fig. 1D) with a calculated *K*_i_ of 1.9 ± 0.2 mM. The concentration of Mg^2+^ did not affect this substrate inhibition (Supplementary Fig. S1). Substrate inhibition could represent a biologically relevant regulatory mechanism (Reed *et al*., 2010), indicating that *Af*UAP1 may be involved in the regulation of UDP-GlcNAc biosynthesis, similar to UAP from *Giardia lamblia* (Bulik et al., 2000).

**Table 1:** Kinetic parameters for *Af*UAP1 compared with *Hs*UAPs (AGX1 and AGX2)

	GlcNAc-1P	UTP			
	*K*_m_ (μM)	*K*_m_ (μM)	*V*_max_ (μM s^−1^)	*k*_cat_ (s^−1^)	*k*_cat_*/K*_m_ (μM^−1^ s^−1^)
*Af*UAP1	34 ± 3	21 ± 2	0.044 ± 0.001	2.2 ± 0.3	0.1048
AGX1 (Peneff *et al*., 2007)	5.3	53	NR	63.6	1.2
AGX2 (Peneff *et al*., 2007)	6	49	NR	68.6	1.4

The results are the mean ± SD for three determinations. NR, not reported.

### *uap1* is an essential gene for *A. fumigatus* growth

**Figure 2:**
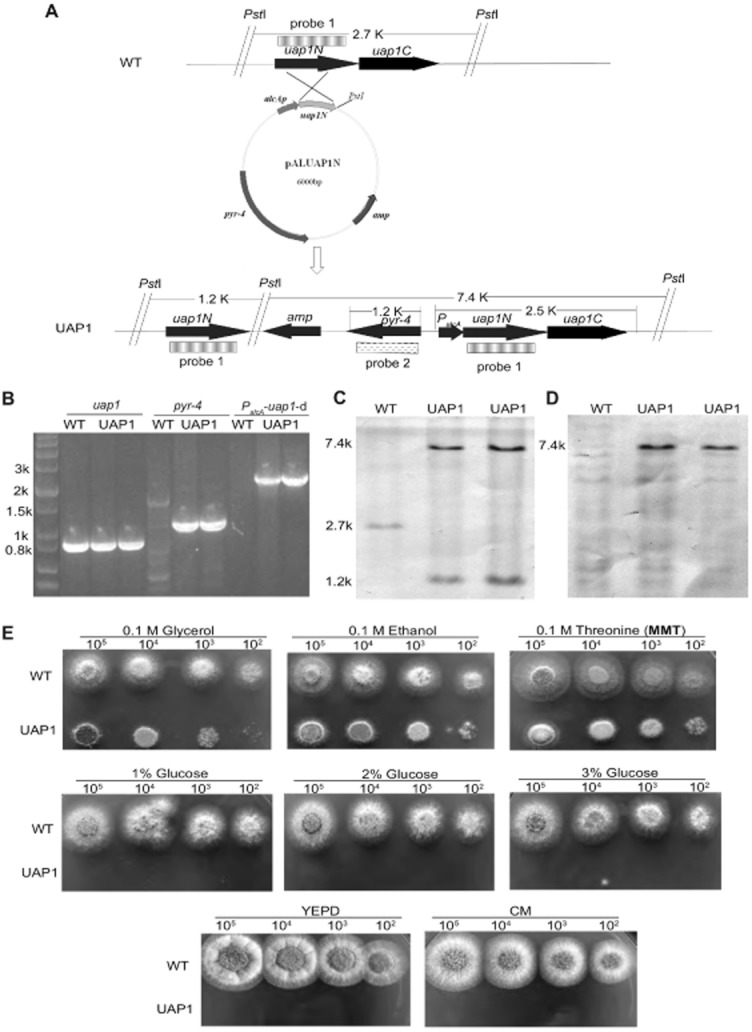
Generation of a conditional inactivation *uap1* mutant.A. Schematic diagram of the strategy towards the conditional mutant.B. PCR confirmation of the *uap1* mutant using primer pairs of P7 & P8, P11 & P12, P9 & P10 to amplify the *uap1* gene, the *N. crassa* *pyr-4* gene and the fragment of P_*alcA*_- downstream of *uap1* respectively.C. Southern blot using an 880 bp fragment of the *uap1* gene as a probe (marked as probe 1 in Fig. 2A).D. Southern blot using a 1.2 kb HindIII internal fragment of the *N. crassa* *pyr-4* gene as a probe (marked as probe 2 in Fig. 2A).E. Growth of *A. fumigatus* strains on solid MM supplemented with 0.1 M glycerol, 0.1 M ethanol, 0.1 M threonine or 1%, 2%, 3% glucose, YEPD or CM, using serial dilutions of 10^5^–10^2^ conidia.

Initial attempts to construct a deletion mutant by replacing the *uap1* gene with the *pyrG* resistance cassette failed (86 transformants screened). Subsequently, a conditional inactivation mutant was constructed by replacing the native promoter of the *uap1* gene with the *Aspergillus nidulans alcA* promoter (P*_alcA_*), a tightly regulated promoter induced by ethanol, glycerol or threonine, repressed by glucose and completely repressed on YEPD medium (Waring *et al*., 1989; Romero *et al*., 2003). The plasmid pALuap1N that contains the *Neurospora crassa pyr-4* gene and P*_alcA_* fused to a 3′ truncated version of the *uap1* gene, was used to transform *A. fumigatus* CEA17 to generate a conditional mutant by homologous recombination (Fig. 2A). Two transformants, confirmed as the correct mutants, were named as UAP1. PCR analysis revealed a 1217 bp fragment of the *N. crassa pyr-4* gene and a 2548 bp fragment of P*_alcA_*–*uap1* fusion was amplified from the UAP1 strain but not from the wild type (WT, Fig. 2B). In Southern blot confirmation when an 880 bp fragment of the *uap1* gene was used as a probe, the expected 2.7 kb fragment was found in the WT, while the expected 1.2 kb and 7.4 kb fragments were detected in the UAP1 strain (Fig. 2C). When a 1.2 kb fragment of the *N. crassa pyr-4* gene was used as probe, an expected 7.4 kb fragment was detected in the UAP1 strain only (Fig. 2D). These results confirmed that the promoter of the *uap1* gene was replaced by the P*_alcA_* promoter in the UAP1 strain.

Growth of the UAP1 strain was induced on solid minimal medium (MM) containing 0.1 M glycerol, 0.1 M ethanol or 0.1 M threonine (MMT) after 36 h at 37°C, and completely inhibited on YEPD, CM or MM containing 1–3% glucose (Fig. 2E). Thus, expression of *uap1* is required for *A. fumigatus* viability.

### *uap1* is important for cell wall ultrastructure, integrity and synthesis

**Figure 3:**
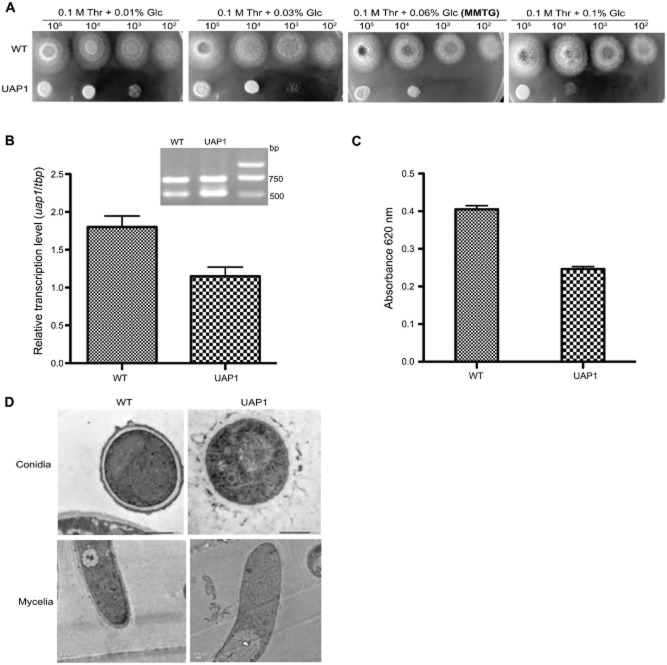
The UAP1 strain under repressive growth conditions.A. Growth on solid MM supplemented with 0.1 M threonine and 0.01%, 0.03%, 0.06% or 0.1% glucose, using serial dilutions of 10^5^–10^2^ conidia.B. Semi-quantitative RT-PCR to amplify the *tbp* and *uap1* gene. The RT-PCR products were separated on agarose gel (inset) and the relative transcription level was calculated based on the band intensity.C. Enzyme activity of the UAP1 strain grown under suppression condition. Intracellular proteins were extracted from mycelium and the enzyme activity was detected by Biomol green assay coupled with pyrophosphatase.D. TEM spore and mycelia morphology of strains grown in solid and liquid MMTG medium at 37°C for 36 h. Conidia (upper panel) and mycelia (lower panel) were fixed and examined with an H-600 electron microscope (Hitachi). Bar = 0.5 μm.

When the UAP1 strain was inoculated on MM supplemented with varying concentrations of threonine and glucose, growth was affected by the ratio of the latter two components. On MM containing 0.1 M threonine and 0.01–0.1% glucose, growth of the UAP1 strain was partially inhibited (Fig. 3A). To investigate the function of *Af*UAP1, MM with 0.1 M threonine and 0.06% glucose (MMTG) was selected for subsequent analysis. Under this condition, total RNAs were prepared from mycelia and the transcription levels of *uap1* in the UAP1 strain and the WT were examined by semi-quantitative RT-PCR. The transcription of the *uap1* gene was reduced to 64% of the WT (Fig. 3B). Intracellular proteins were extracted from the mycelium and investigated for *Af*UAP1 activity, revealing a 39% reduction in activity of the UAP1 strain compared with the WT strain (Fig. 3C).

Examination of the ultrastructure of the conidia and hyphal cell wall showed that although the conidia in the UAP1 strain have a thicker cell wall, it does not retain melanin on its surface (Fig. 3D, upper panel), suggesting differences in cell wall architecture. In contrast, the hyphae of strain UAP1 had a thinner cell wall (Fig. 3D, lower panel).

**Figure 4:**
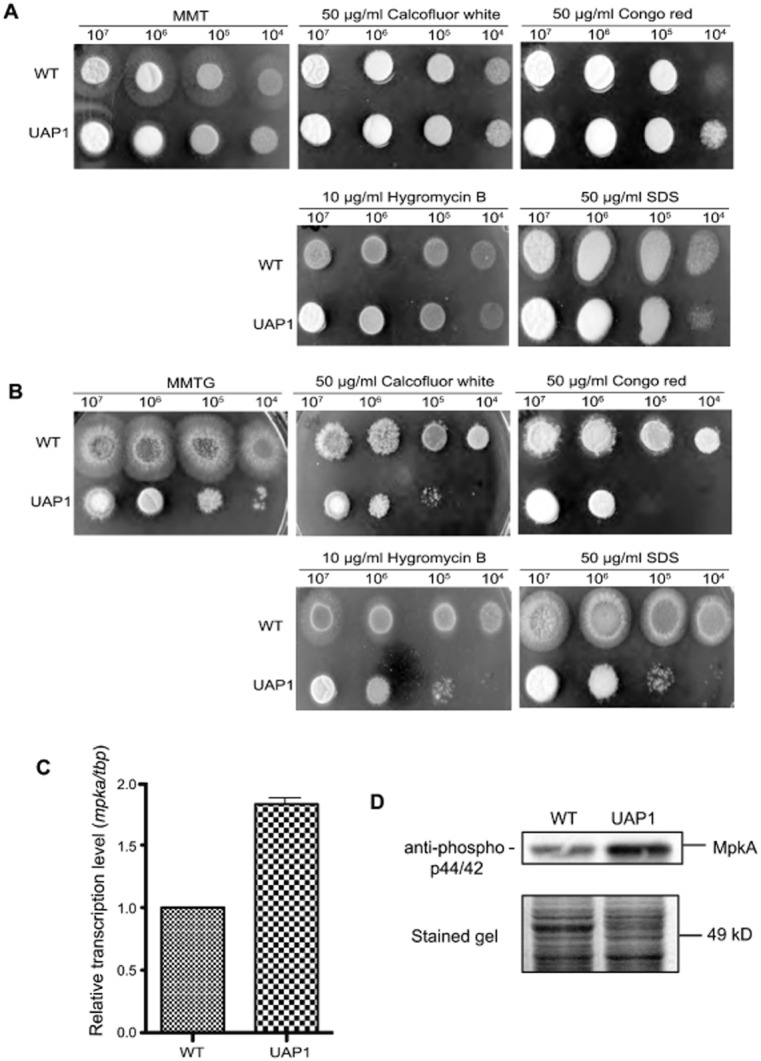
Sensitivity of the UAP1 strain to chemical reagents and phosphorylation of MpkA.A and B. Serial dilutions of conidia from 10^7^ to 10^4^ were spotted on solid (A) MMT, (B) MMTG containing 50 μg ml^−1^ Calcofluor white, 50 μg ml^−1^ Congo red, 50 μg ml^−1^ SDS or 10 μg ml^−1^ hygromycin B. After incubation at 37°C for 48 h, the plates were taken out and photographed.C. Real-time PCRs using primers P17 & P18, P19 & P20 were performed to analyse the transcription levels of MpkA in strains after 36 h cultivation in MMTG. The experiment was repeated three times and the transcription level of WT was set as onefold.D. Phosphorylated MpkA was detected using anti-phospho p44/42 MAPK antibodies. A Coomassie-stained gel is shown as loading control.

We also tested the sensitivity of the UAP1 strain to various chemical reagents. Using growth conditions (MMT media) that induced expression of *uap1*, the UAP1 strain showed no sensitivity to these reagents, similar to the WT (Fig. 4A). However, in media (MMTG) that suppressed induction of *uap1* expression, the UAP1 strain showed an increased sensitivity to Congo red, Calcofluor white, SDS and hygromycin B as compared with the UAP1 strain grown on MMTG plates alone (Fig. 4B), suggesting that reduced expression of the *uap1* led to a defect in cell wall integrity.

To investigate these findings, cell wall contents were analysed (Table 2). Compared with the WT strain, the content of α-glucan and chitin in the mycelial cell wall in the UAP1 strain decreased by 36% and 11% respectively. Although the glycoprotein and β-glucan content in the UAP1 strain were similar to the WT strain, GlcNAc released from these proteins was reduced by 23% while mannose increased by 59%. These results suggest that reduced expression of the *uap1* gene in *A. fumigatus* induced decreased content of α-glucan, chitin and GlcNAc in the cell wall. As Man and GlcNAc are both derived from Fru-6P, the increase of GDP-Man could be the result of diversion of Fru-6P away from UDP-GlcNAc biosynthesis in the UAP1 strain. Furthermore, the increased mannose content in cell wall may represent part of a compensatory mechanism to the weakened cell wall.

**Table 2:** Chemical analysis of the cell wall in MMTG medium

	Alkali soluble					Alkali insoluble	
	Glycoprotein						
Strain	Protein (μg)	GlcNAc(μg)	Gal (μg)	Man (μg)	α-Glucan (μg)	Chitin (μg)	β-Glucan (μg)
WT	194 ± 4	0.22 ± 0.02	1.39 ± 0.13	2.23 ± 0.04	280 ± 29	322 ± 1	1282 ± 20
UAP1	188 ± 5	0.17 ± 0.01	1.25 ± 0.15	3.54 ± 0.39	178 ± 2	288 ± 13	1361 ± 59

Three aliquots of 10 mg lyophilized mycelia were used as independent samples for cell wall analysis, and the experiment was repeated twice. The values shown are microgram of cell wall component per 10 mg dry mycelia (± SD).

A potential response to a defective cell wall is activation of the cell wall integrity (CWI) pathway via activation of the MAP kinase MpkA (Jain *et al*., 2011). We studied the *mpkA* transcription level and MpkA phosphorylation in the UAP1 strain under conditions of *uap1* suppression. Real-time PCR analysis revealed that *mpkA* was induced 1.8-fold (*P* < 0.05 (Fig. 4C). A Western blot with a phospho-specific antibody showed that MpkA was phosphorylated at a higher level in the UAP1 strain than in the WT strain (Fig. 4D), indicating that cell wall defects in the UAP1 strain induced the activation of the MpkA-dependent CWI pathway.

### *Af*UAP1 is required for morphogenesis

**Figure 5:**
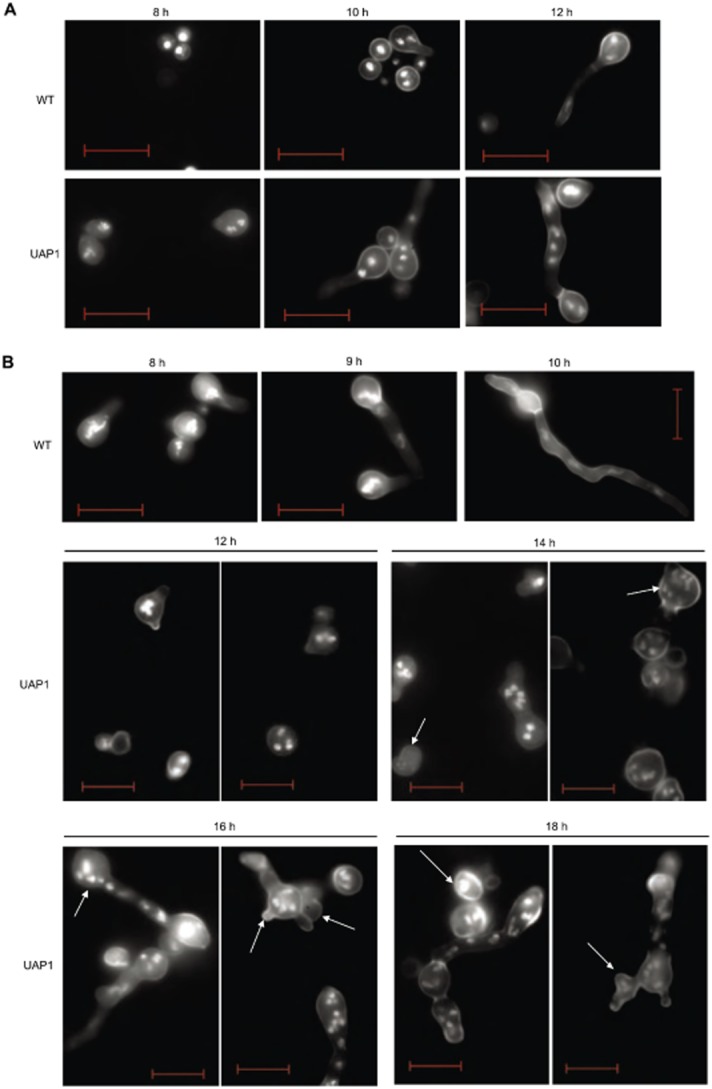
Conidia germination of the UAP1 strain. A total of 10^7^ freshly harvested conidia were inoculated into 10 ml of (A) MMT, (B) MMTG in a Petri dish containing glass coverslips. After incubation at 37°C for specified time, the coverslips with adhering germlings were removed and fixed. Coverslips were then washed with PBS, incubated for 20 min with 1 μg ml^−1^ DAPI, washed with PBS, and then incubated for 10 min with a 10 μg ml^−1^ Calcofluor white, washed again. Germlings were photographed using a fluorescence microscope (Carl Zeiss). Typical photographs are shown. Bar = 10 μm.

The conidia of filamentous fungi undergo a series of ordered morphological events including the switch from isotropic to polar growth, the emergence of a second germ tube and septation (Momany and Taylor, 2000). To study the effect of *Af*UAP1 activity on hyphal development, conidia from the UAP1 strain were allowed to adhere to glass coverslips during incubation under conditions of *uap1* induction or suppression. Coverslips with adhering germlings were fixed, stained with 4′,6-diamidino-2-phenylindole (DAPI) and Calcofluor white and examined under a fluorescence microscope. With *uap1* induction (MMT), 95% of the UAP1 strain displayed germination after 10 h incubation (Fig. 5A), while only 89% of the WT strain showed germination after 12 h incubation, demonstrating that the UAP1 strain displayed rapid conidial germination, approximately 2 h earlier in initiation. No abnormal polarity was observed for the UAP1 strain under this condition. With *uap1* suppression (MMTG), the WT had 89% germination at 9 h and 100% germination after 10 h, whereas the UAP1 strain had only 65% germination at 14 h, and still had 11% ungerminated cells even after 16 h incubation. Apart from 5 h delayed germination, the UAP1 strain germ tubes formed anomalous bubbles from 14 h, displayed multiple germ tubes on each basal cell and ballooned or highly branched hyphal tips (Fig. 5B, white arrow). These results suggest that UAP1 is involved in morphogenesis of *A. fumigatus*.

### The AGX1 and *Af*UAP1 active sites possess exploitable differences

**Figure 6:**
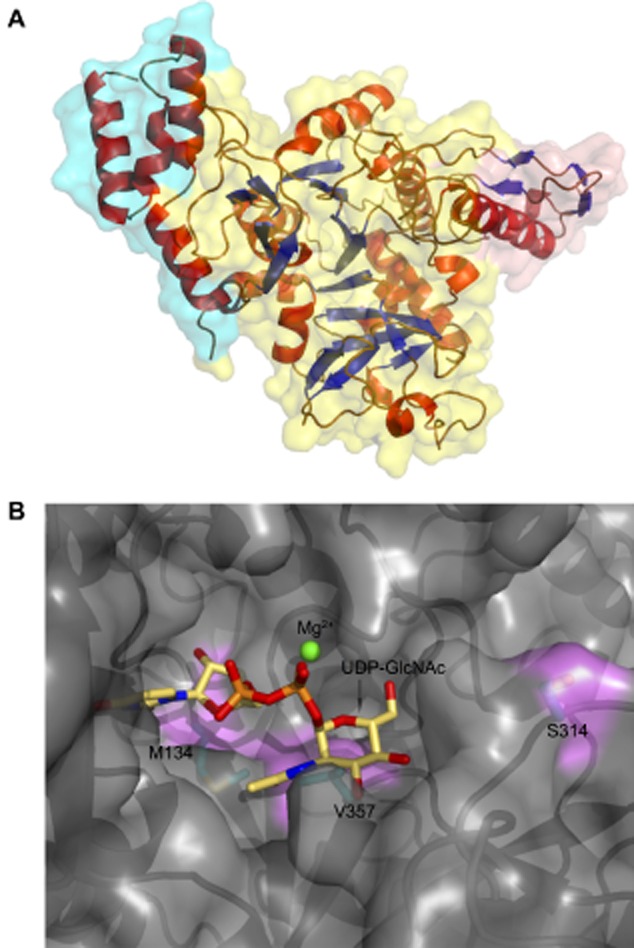
Crystal structure of *Af*UAP1.A. A structural overview of *Af*UAP1. The N-terminal domain is coloured in cyan, the central domain is coloured in yellow and the C-terminal domains is coloured in salmon. Secondary structure elements of each domain are coloured red (helices) and blue (strands).B. Close-up view of the *Af*UAP1 active site. A molecular surface of *Af*UAP1 is shown, conserved residues with AGX1 are coloured in grey, difference residues are coloured in violet, side-chains are shown in cyan. A model of UDP-GlcNAc obtained by superposition with the AGX1-UDP-GlcNAc complex (PDBID 1JV1; Peneff *et al*., 2007) is shown as sticks with yellow carbon atoms. The predicted position of Mg^2+^ was obtained by superposition with the UGPase (PDBID 3JUK; Kim *et al*., 2010) and is shown as green sphere.

AGX1 and *Af*UAP1 share 59% identical at the amino acid sequence level. To explore potential exploitable differences in the active site, the crystal structure of *Af*UAP1 was determined to 2.1 Å resolution (Table 3, Fig. 6A). Similar to the structures of other eukaryotic UAPs reported to date (Peneff *et al*., 2007; Maruyama *et al*., 2002), the overall structure of *Af*UAP1 reveals a three-domain fold with the central catalytic domain flanked by two domains of unknown function (Fig. 6A). The N-terminal domain of *Af*UAP1 consists of four α-helices and six β-strands. The central domain consists of 11 β-strands and 12 helices adopting the Rossmann fold (Rao and Rossmann, 1973). The C-terminal domain consists of four short β-strands and a long helix that connects to the central domain (Fig. 6A).

**Table 3:** Details of X-ray diffraction, data collection and structure refinement

	*Af*UAP1
Resolution (Å)	20.0 (2.15–2.08)
Space group	P2_1_2_1_2_1_
Unit cell	
*a* (Å)	55.6
*b* (Å)	139.7
*c* (Å)	144.7
No. of reflections	246191
No. of unique reflections	64787
*I*/σ (*I*)	11.18 (2.06)
Completeness (%)	98.62 (99.7)
Redundancy	3.8 (3.8)
*R*_merge_ (%)	11.3 (73.1)
RMSD from ideal geometry	
Bond distances (Å)	0.018
Bond angle (°)	2.0
*R*_work_ (%)	21.2
*R*_free_ (%)	26.5
No. of residues	924
No. of water mol.	332
<B> protein(Å^2^)	20.6

Values in parenthesis are for the highest resolution shell. All measured data were included in structure refinement.

*Af*UAP1 has a large active-site cleft, with its wall being formed by the N- and C-terminal domains. Several of the amino acids (e.g. equivalents of Thr140, Arg141 and Lys148 in *Af*UAP1) shown to co-ordinate the phosphates of nucleotides/phospho-sugars in complexes with other UAP1 enzymes have been probed by mutagenesis and shown to be essential for substrate binding/catalysis (Mio *et al*., 1998; Peneff *et al*., 2007; Maruyama *et al*., 2002). There are two homologous forms of UAP in *Arabidopsis,* GlcNAc1pUT-1 and GlcNAc1pUT-2, showing different substrate specificities. Both enzymes convert GlcNAc-1P and GalNAc-1P to UDP-GlcNAc and UDP-GalNAc, whereas only GlcNAc1pUT-2 converts Glc-1P to UDP-Glc (Yang *et al*., 2010). Homology modelling using AGX1 as a template revealed that the residues Glu335, Asn360, Gly320 and Asn253 of GlcNAc1pUT-1 were involved in recognition of the hexosamine moiety (Yang *et al*., 2010). However, because all these residues are identical in GlcNAc1pUT-2, AGX1 and *Af*UAP1, they are unlikely contribute to UAP substrate specificity. The models also suggested that the loop region between residues Pro312 and Gly317 in GlcNAc1pUT-2 might account for its broader substrate specificity (Yang *et al*., 2010). However, in the *Af*UAP1 structure this region is positioned far from the sugar moiety.

Apart from UAP, there are other pyrophosphorylases, such as UDP-glucose pyrophosphorylase (UGPase, A- and B-type) and UDP-sugar pyrophosphorylase (USPase), that have relatively high structural homology to UAP and carry out similar reactions with sugar-1P and UTP (Kleczkowski *et al*., 2011). Compared with the structures of UGPase [PDB ID: 2ICY (McCoy *et al*., 2007); 3JUK (Kim *et al*., 2011)] and USPase [PDB ID: 3OH2 (Dickmanns *et al*., 2011)] *Af*UAP1 has a larger sugar binding pocket that can accommodate the bulky *N*-acetyl moiety of GlcNAc-1P whereas the sugar binding pockets of the other pyrophosphorylases are too small to accommodate this substrate.

Compared with the human enzyme (Peneff *et al*., 2007), the *Af*UAP1 active site possesses three substitutions: Met134, Ser314 and Val357 (Fig. 6B), corresponding to Leu108, Pro288 and Ala329 in AGX1 respectively. These differences might account for the differences in sugar phosphate specificity, with *Af*UAP1 being more selective for GlcNAc-1P while AGX1 can utilize both GlcNAc-1P and GalNAc-1P as substrates. Furthermore, these differences in the active site between the fungal enzyme and its human orthologue suggest that it may be possible to identify selective inhibitors of *Af*UAP1.

### Conclusion

Due to the increasing number of immunocompromised patients, the morbidity and mortality of invasive fungal infections (IFIs) are continuously high (Kousha *et al*., 2011). However, current antifungal drugs have not kept pace with this escalating medical demand. The numbers of antifungal drug families for IFIs remain limited to polyenes, azoles and echinocandins (Denning and Hope, 2010). Both polyenes and azoles are effective in clinical treatment but also have numerous adverse effects and toxicity (Masia Canuto and Gutierrez Rodero, 2002; Snelders *et al*., 2008; Kaji *et al*., 2009; Moen *et al*., 2009) as they target the cell membrane that is also present in mammalian cells. Only the echinocandin-class drugs specifically target the fungal cell wall but resistance has arisen recently (Perlin, 2007; Niimi and Niimi, 2009). Therefore, new cell wall drug targets are required.

UAP is the final enzyme involved in the biosynthesis of UDP-GlcNAc, the direct precursor in the synthesis of cell wall chitin. Here we have genetically validated UAP as an attractive antifungal drug target in pathogenic fungi. The essentiality of the *uap1* gene in *A. fumigatus* was demonstrated with a conditional inactivation mutant, which was unable to grow under conditions of complete suppression (Fig. 2D). This suggests that there is no alternative metabolic route to synthesize UDP-GlcNAc in *A. fumigatus*. Under conditions of partial gene suppression, to allow for limited growth and phenotypic analysis, the mutant displayed a defective cell wall (Fig. 4B), decreased chitin and GlcNAc content (Table 2), presumably as a direct consequence of the decreased pools of the UDP-GlcNAc precursor. The MpkA-mediated cell wall integrity signalling pathway plays an important role in maintaining cell wall biogenesis under stress conditions. The increased level of MpkA phosphorylation in the UAP1 strain is consistent with activation of this pathway. *Af*UAP1 is also essential for morphogenesis as shown by the mutant displaying delayed germination and abnormal morphology under conditions of *uap1* suppression (Fig. 5B). This is in agreement with reduced UDP-GlcNAc levels leading to reduced levels of cell wall chitin synthesis, which is essential and highly dynamic during morphogenesis.

To develop the next generation of non-toxic antifungals, it is necessary to effectively target pathogen enzymes rather than their human orthologues. Here, the crystal structure of *Af*UAP1 revealed three non-conserved residues at the active site (Fig. 6B), which may account for the stricter substrate specificity of *Af*UAP1 compared with the human orthologues. These differences could be exploitable for structure-guided design of fungal-specific inhibitors. Interestingly, substrate inhibition of *Af*UAP1 by UTP was also detected (Fig. 1D), which may represent a possible mechanism for regulation of the UDP-GlcNAc biosynthetic pathway in addition to the feedback inhibition by UDP-GlcNAc reported for glucosamine-fructose-6-phosphate aminotransferase (Kornfeld, 1967; Winterburn and Phelps, 1971), the first enzyme in this pathway.

In conclusion, by combination of genetic and structural approaches we have validated *Af*UAP1 as a potential antifungal drug target. The structural insights reported here will aid the future discovery and design of novel antifungal compounds.

## Experimental procedures

### Reagents, strains and growth conditions

*N*-acetylglucosamine-1-phosphate (GlcNAc-1P), glucose-1-phosphate (Glc-1P), galactose-1-phosphate (Gal-1P), glucosamine-1-phosphate (GlcN-1P) and GlcNAc-6P were purchased from Sigma-Aldrich, mannose-1-phosphate (Man-1P) was purchased from Carbosynth, *N*-acetylgalactosamine-1-phosphate (GalNAc-1P) was a kind gift from Prof. Peng George Wang, Shandong University, China.

*Aspergillus fumigatus* strain YJ-407 (CGMCC0386) was maintained on potato glucose (2%) agar slants (Xia *et al*., 2001) and strain CEA17 (Weidner *et al*., 1998) was propagated at 37°C on YGA (0.5% yeast extract, 2% glucose, 1.5% Bacto-agar) with the addition of 5 mM uridine and uracil (d'Enfert, 1996). The P*_alcA_* was induced by growth on MM (Armitt *et al*., 1976) supplemented with 0.1 M glycerol, 0.1 M threonine or 0.1 M ethanol as carbon sources. YEPD (2% yeast extract, 2% glucose and 0.1% peptone) medium and complete medium (CM; Cove, 1966) was utilized to repress the P*_alcA_*. Strains were grown in liquid medium at 37°C, with shaking at 200 r.p.m. After approximately 3 days, mycelia were harvested, washed with distilled water, frozen in liquid nitrogen, and then ground using a mortar and pestle. The powder was stored at −70°C for DNA, RNA and protein extraction.

Conidia were prepared by propagating *A. fumigatus* strains on solid medium for 48 h at 37°C. Spores were collected, washed twice with PBS containing 0.1% Tween 20 and resuspended in distilled water. The concentration of spores was verified by haemocytometer and viable counting.

### Cloning of *A. fumigatus* *uap1*

The *A. fumigatus uap1* gene contains three exons interrupted by two introns at nucleotide positions 724–786 and 1522–1595. Initially, the entire coding region of this gene was obtained by PCR from *A. fumigatus Af293* genomic DNA (kindly provided by Jean-Paul Latgé, Institut Pasteur, France), using the forward primer P1 (5′-CTCAATTGATGGCCGTAGCTATCAAAGAGACAGTC-3′) and the reverse primer P2 (5′-CTGCGGCCGCTTATTCTTCTTTCTCAATGAAGGCTGGTGCTTTG-3′), containing a recognition sequence (underlined) for MfeI (MunI) and NotI respectively. This was cloned into the pSC-B vector (Stratagene) and the introns were subsequently removed by PCR-based deletion mutagenesis using the forward primer P3 (5′-GGCAAGATCTTGATGGAGAGCAAGTTCAAG*GTCGCTGTTGCTCCTGATGGAAACGGTGG*-3′), consisting exon I 3′ end (underlined) and exon II 5′ end (italic); and the reverse primer P4 (5′-*GGCCTTTTAGGAATTCAAGACCCTCTCCGCC*ATAGCTGATCAGTGGAGATACTTCAACAC-3′), consisting of exon III 5′ end (italic) and exon II 3′ end (underlined). This mutagenesis regenerated the *uap1* open reading frame (Accession No. XM_741621, NCBI), which was subsequently digested with MfeI and NotI and subcloned into an EcoRI–NotI-digested pGEX-6P-1 vector (GE Healthcare). This vector encodes a glutathione-*S*-transferase (GST) tag followed by a PreScission protease cleavage site. Finally, vector polylinker sequences and the *uap1* sequence encoding amino acid residues 1–27 were removed by site-directed mutagenesis using the forward and reverse mutagenesis primers P5 (5′-CTGGAAGTTCTGTTCCAGGGGCCCTCTGCTGAAGAGTTCCAGCAGCTTAG-3′) and P6 (5′-CTAAGCTGCTGGAACTCTTCAGCAGAGGGCCCCTGGAACAGAACTTCCAG-3′), respectively, resulting in the final expression plasmid pGEX-6P-1-*Af*UAP1 Δ1–27.

### Construction of the *uap1* conditional inactivation mutant

Plasmid pAL3 (Waring *et al*., 1989) containing the P*_alcA_* and the *N. crassa pyr-4* gene as a fungal selectable marker was used to construct a suitable vector allowing the replacement of the native promoter of the *uap1* with the P*_alcA_*. An 880 bp fragment from −40 to +840 of the *uap1* genomic DNA sequence was amplified with primers P7 (5′-GGGGTACCCCCTCTTCGTCTGATACGCTC-3′), containing a KpnI site and P8 (5′-GCTCTAGAGGATGTAAGAAGGGCCTGGTAG-3′), containing an XbaI site. The PCR-amplified fragment was cloned into the expression vector pAL3 to yield pALuap1N and confirmed by sequencing. The pALuap1N was used to transform *A. fumigatus* strain CEA17 by PEG-mediated fusion of protoplasts (Langfelder *et al*., 2002) and positive transformants were selected by uridine/uracil autotrophy. The transformants were confirmed by PCR and Southern blot analysis. For PCR analysis, three pairs of primers (P7 & P8, P9 & P10 and P11 & P12) were utilized. Primers P7 and P8 were used to amplify an 880 bp fragment of the *uap1* gene. P9 (5′-TCGGGATAGTTCCGACCTAGGA-3′) and P10 (5′-TCCGCATTGCGTAAGGTTGC-3′) were used to amplify a 2.54 kb fragment from the P*_alcA_* to a downstream flanking region of the *uap1* gene. Primers P11 (5′-AAACGCAAATCACAACAGCCAAC-3′) and P12 (5′-CTATGCCAGACGCTCCCGG-3′) were used to amplify the *N. crassa pyr-4* gene. For Southern blotting, genomic DNA was digested with PstI, separated by electrophoresis, and transferred to a nylon membrane (Zeta-probe^+^, Bio-Rad). The 880 bp fragment of the *uap1* gene and a 1.2 kb HindIII fragment of the *N. crassa pyr-4* gene from pAL3 were used as probes. Labelling and visualization were performed using the DIG DNA labelling and detection kit (Roche Applied Science) according to the manufacturer's instructions.

### Semi-quantitative RT-PCR

Total RNAs from the spores cultured in liquid MM supplemented with 0.1 M threonine and 0.06% glucose (MMTG) were extracted using Trizol reagent (Invitrogen). Complementary DNA synthesis was performed with 5 μg of RNA using the SuperScript-First-Strand Synthesis System (Fermentas). Primers P13 (5′-CCACCTTGCAAAACATTGTT-3′) and P14 (5′-TACCAGAAAGCACAGGATAG-3′) were used to amplify a 515 bp fragment containing the intron and exon of the *tbp* gene (encoding TATA-binding protein). Primers P15 (5′-AGTTCAAGGTCG CTGTTGCT-3′) and P16 (5′-GGAGATACTTCAACACCAAC-3′) were used to amplify a 750 bp fragment containing the intron and exon of the *uap1*. The *tbp* and *uap1* fragments were amplified in one tube with primer pairs (P13 & P14 and P15 & P16) in a 1:2 ratio. Twenty-eight PCR cycles (94°C for 45 s, 58°C for 50 s and 72°C for 50 s) were performed. The products were loaded onto an ethidium bromide-stained 1.2% agarose gel and quantification of the bands was performed by Quantity One (Bio-Rad). Band density ratios were calculated by dividing normalized values of *uap1* by *tbp*.

### Analysis of the *uap1* conditional mutant

To monitor the cell wall structures, the conidia and mycelia grown in solid and liquid MMTG or MM containing 0.1 M threonine (MMT) were fixed and examined with an H-600 electron microscope as described by Li *et al*. (2007).

To test the sensitivity to chemical reagents, serial dilutions of conidia from 10^7^ to 10^4^ were spotted on MMT or MMTG plates containing 50 μg ml^−1^ of Calcofluor white, 50 μg ml^−1^ of Congo red, 50 μg ml^−1^ of SDS or 10 μg ml^−1^ of hygromycin B respectively. After incubation at 37°C for 48 h, the plates were taken out and photographed.

For the chemical analysis of the cell wall, conidia were inoculated into 100 ml MMTG liquid medium at a concentration of 10^6^ conidia ml^−1^ and incubated at 37°C with shaking at 200 r.p.m. for 36 h. The mycelia were harvested, washed with deionized water and stored at −80°C. The cell wall components were isolated and assayed as described previously (Fang *et al*., 2009). Three independent samples of lyophilized mycelia pad were used for cell wall analysis, and the experiment was repeated twice.

For examination of nuclei, septa and cell wall staining at the germination stage, 10 ml of MMT or MMTG medium was inoculated with 10^7^ freshly harvested conidia in a Petri dish containing glass coverslips. After incubation at 37°C for the time indicated, the coverslips with adhering germlings were removed and fixed in 4% formaldehyde in PBS which contain 0.2% Triton X-100 for 30 min. The coverslips were then washed with PBS, incubated for 20 min with 1 μg ml^−1^ DAPI (Sigma). After PBS wash the coverslips were then incubated for 10 min with 10 μg ml^−1^ Calcofluor white (Sigma), washed again, then the germlings were photographed using a fluorescence microscope (Carl Zeiss).

### Detection of MpkA

To detect the transcription level of MpkA, total RNAs and cDNA were prepared as described above. Primers P17 (5′-ACCAAAGCTATCGACGTGTG-3′) and P18 (5′-AGTCCCGGCCTTTGAAGAAA-3′) were used to amplify a 78 bp fragment of the *mpkA* (AFUA_4G13720), and primers P19 (5′-CCACCTTGCAAAACATTGTT-3′) and P20 (5′-TACTCTGCATTTCGCGCATG-3′) were used for an 80 bp fragment of *tbp*. To exclude contamination of cDNA preparations with genomic DNA, primers were designed to amplify regions containing one intron in the gene. PCR reactions were performed with a LightCycler® (Roche) instrument loaded with eight strip PCR tubes (Roche). Each PCR reaction mixture (20 μl) contained 5 μl sample cDNA, 10 μl SYBR *Premix Ex Taq*™ from SYBR *Premix Ex Taq*™ Kit (TAKARA), 4.2 μl ddH_2_O and 0.2 μM of each pair of primers. Thermal cycling conditions were 95°C for 50 s, followed by 40 cycles of 95°C for 5 s, 60°C for 20 s. Real-time PCR data were acquired using Sequence Detection software. Samples isolated from different strains were tested in triplicate.

To detect the phosphorylation level of MpkA, the mycelia were incubated, harvested and ground as previously described. The powdered mycelia were suspended in lysis buffer (200 mM Tris-HCl, pH 8.0, 20 mM EDTA, 1 mM phenylmethanesulphonyl fluoride) and incubated on ice for 20 min. Proteins in the supernatant were collected by centrifugation (16 000 *g* at 4°C for 10 min). Protein concentration was determined by the Lowry method (Bensadoun and Weinstein, 1976; Peterson, 1977). Thirty micrograms of cellular proteins was separated by 10% SDS-PAGE and transformed to PVDF membrane (Millipore, USA). The phosphorylation of MpkA was examined using anti-phospho-p44/42 MAPK antibody (Cell Signaling Technology, USA). The primary antibody was detected using Anti-Rabbit IgG Horseradish peroxidase (HRP) Conjugate (Promega) and Enlight^TM^ reagents (Engreen Biosystem, China).

### Expression and purification of *Af*UAP1

The N-terminally truncated pGEX-6P-1-*Af*UAP1 Δ1–27 plasmid was transformed into *E. coli* BL21 (DE3) pLysS and a single colony inoculated into 100 ml of Luria–Bertani (LB) medium containing 0.1 mg ml^−1^ ampicillin and incubated at 37°C overnight with shaking at 200 r.p.m. Ten millilitres of the overnight culture was used to inoculate 1 l LB medium and grown to an OD_600_ of 0.6. Expression of the GST fusion protein was induced at room temperature with 250 μM of IPTG (isopropyl-β-d-thiogalactopyranoside) and the cultures incubated for a further 20 h. The cells were then harvested by centrifugation at 3500 *g* for 30 min, resuspended in 25 ml of lysis buffer (25 mM Tris-HCl, 150 mM NaCl, pH 7.5 containing 10 mg ml^−1^ DNase, 0.5 mg ml^−1^ lysozyme and a tablet of protease inhibitor cocktail) (Bensadoun and Weinstein, 1976) and lysed by sonication on ice. The cell lysate was centrifuged at 40 000 *g* for 30 min to remove cell debris and the supernatant was incubated with pre-washed glutathione sepharose beads (GE Healthcare) at 4°C on a rotating platform for 2 h. The GST-*Af*UAP1 fusion protein was isolated by centrifugation and washed with buffer (25 mM Tris-HCl, 150 mM NaCl, pH 7.5). The GST tag was cleaved overnight with PreScission protease (50 μg protease per ml of beads). The cleaved protein was filtered from the beads, concentrated to 5 ml and loaded onto a Superdex 200 column (Amersham Bioscience) equilibrated with buffer and eluted at a flow rate of 1 ml min^−1^ in the same buffer. The fractions were verified by SDS-PAGE. Pure fractions were pooled and concentrated to 20 mg ml^−1^ using a 10 kDa cut-off Vivaspin concentrator (GE Healthcare).

### Enzyme kinetics

*Af*UAP1 steady-state kinetics was studied under linear conditions with no more than 10% of substrate conversion. The activity of *Af*UAP1 for the forward reaction was determined as described by Mok and Edwards (2005a). Briefly, the assay was performed in triplicate in a 96-well plate at 37°C with each well containing 50 mM Tris-HCl, pH 7.5, 10 mM MgCl_2_, 10% (v/v) glycerol, 1 mM DTT, 0.4 units of pyrophosphatase, 20 nM of *Af*UAP1 and varying concentrations of GlcNAc-1P and UTP in a final volume of 100 μl. The reaction was incubated for 10 min and terminated by the addition of 100 μl Biomol green [0.03% w/v malachite green, 0.2% w/v ammonium molybdate and 0.5% (v/v) Triton X-100 in 0.7 N HCl] followed by 20 min incubation at room temperature. The absorbance intensity was measured at 620 nm using a Spectra max 340 PC (Molecular Devices) and data were analysed with non-linear regression analysis using GRAFIT 5 (Leatherbarrow, 2001) with the default equations for first-order reaction rates and Michaelis-Menten steady-state kinetics.

Catalysis of the reverse reaction was analysed by High Performance Anion Exchange Chromatography (HPAEC, Dionex) using conditions adapted from Tomiya *et al*. (2002). The eluent was monitored at 260 nm for the presence of UTP and UDP-GlcNAc, with peaks assigned by comparison to the commercial standards. The 100 μl reaction contained 200 μM UDP-GlcNAc, 100 μM pyrophosphate, 10 mM MgCl_2_, 1 mM DTT, 2% glycerol and 20 nM *Af*UAP1 in 10 mM Tris-HCl pH 7.5. This was incubated for 30 min, alongside a negative control without the enzyme, and the reaction terminated by the addition of 10 μl of 100 mM NaOH prior to analysis.

Different phosphosugars: Glc-1P, Gal-1P, GlcN-1P, Man-1P, GalNAc-1P and GlcNAc-6P, were used to investigate substrate specificity using the Biomol green assay described above with GlcNAc-1P as a positive control. 2 mM UTP and 1.6 mM, 2.5 mM, 5 mM phosphosugars were used with 100 nM of *Af*UAP1.

For the *in vivo Af*UAP1 activity assay, ground frozen powder from different strains was dissolved in 50 mM Tris-HCl pH 7.5 and placed on ice for 30 min. Intracellular proteins were collected by centrifugation and protein concentration was determined by Lowery method same as before. *Af*UAP1 activity was determined as described previously (Mok and Edwards, 2005a,b).

### Crystallization, data collection and structure determination

*Af*UAP1 at a concentration of 20 mg ml^−1^ stock solution was used for crystallization trials using the sitting drop method. Each drop contained an equal volume of 0.6 μl of protein and 0.6 μl of reservoir solution (mother liquor). Crystals grew after 3 days in a mother liquor of 0.2 M sodium acetate trihydrate and 20% PEG3350 and were cryoprotected in this solution supplemented with 10% glycerol. Data were collected at the European Synchrotron Radiation Facility (ESRF) (Grenoble, France) and processed with the HKL suite (Otwinowski and Minor, 1997). The structure was solved by molecular replacement using MOLREP (Vagin and Teplyakov, 1997) with the *Ca*UAP1 structure (PDB ID 2YQS; Maruyama *et al*., 2002) as the search model. REFMAC (Murshudov *et al*., 1997) was used for further refinement and iterated with model building using COOT (Emsley and Cowtan, 2004). Images were produced with PyMol (DeLano, 2004). The atomic co-ordinates and structure factors of *Af*UAP1 have been deposited in the Protein Data Bank with accession code 4BMA.
