# Supplementary material for: Genetic and structural validation of Aspergillus fumigatus UDP-N-acetylglucosamine pyrophosphorylase as an antifungal target
Source: Mol Microbiol. 2013 Jul 5;89(3):479–93. doi: 10.1111/mmi.12290 (PMC3888555; doi:10.1111/mmi.12290)
Supplement: Supplementary file 1 [file mmi0089-0479-sd1.pdf]

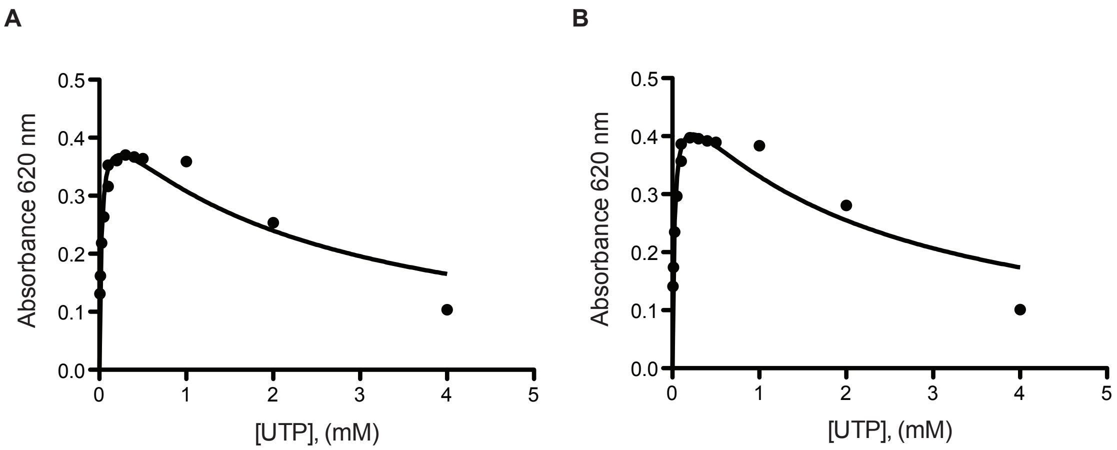

Supplementary Figure S1: Effect of Mg^{2+} on the UTP substrate inhibition. 0.00625-4 mM UTP and 100 μM GlcNAc-1P in the presence of **A.** 1 mM MgCl2, **B.** 20 mM MgCl2 were used in the assay and the products were analyzed in a colorimetric Biomol green assay coupled with pyrophosphatase, giving K_i of 2.4 ± 0.4 mM and K_i of 2.2 ± 0.4 mM, respectively.
